# Influence of testosterone replacement therapy on metabolic disorders in male patients with type 2 diabetes mellitus and androgen deficiency

**DOI:** 10.1186/s40001-014-0056-6

**Published:** 2014-10-23

**Authors:** Shota Janjgava, Tamar Zerekidze, Lasha Uchava, Elene Giorgadze, Ketevan Asatiani

**Affiliations:** National Institute of Endocrinology, 2/6 Ljubljana Street, Tbilisi, 0159 Georgia; Department of Endocrinology, Tbilisi State University, Tbilisi, 0140 Georgia

**Keywords:** Free testosterone, HbA1c, Lipid profile, BMI

## Abstract

**Background:**

Multiple epidemiological studies have shown that low testosterone levels are associated with and predict the future development of type 2 diabetes mellitus and the metabolic syndrome.

The aim of our study was to show the influence of testosterone replacement therapy on obesity, HbA1c level, hypertension and dyslipidemia in patients with diabetes mellitus and androgen deficiency.

**Methods:**

One hundred and twenty-five male patients with diabetes mellitus were screened; 85 subjects aged 41 to 65 years, with BMI from 27.0 to 48.0 kg/m^2^, were randomized in a placebo-controlled study. They also underwent a routine physical examination and selected by free testosterone examination. We divided patients into two groups: 1) treatment group, where we used diet, physical activity, patient’s antidiabetic therapy and testosterone replacement therapy; 2) placebo group, where we used diet, physical activity, patient’s antidiabetic therapy and placebo.

**Results:**

After 6 months of treatment we repeated the diagnostic assessments: lipid profile was improved in both groups but in first group it was clinically significant. Free testosterone level increased in all groups, but in group I was clinically significant. HbA1c decreased in both groups, but in group I we obtained the best result. Leptin level after treatment was approximately the same in both groups. Also, blood pressure was reduced in both groups but results were similar.

**Conclusions:**

Our study demonstrated that it is possible to break this metabolic vicious circle by raising testosterone levels in diabetic men with androgen deficiency. Re-instituting physiological levels of testosterone, as the study has shown, has an important role in reducing the prevalence of diabetic complications.

## Background

Over the past few decades, obesity and diabetes mellitus have become a global health challenge. Between 1980 and 2004, the prevalence of obesity increased from 15 to 33% in the United States - a pattern mirrored across the world [1]. Interlinked with this, the number of people with type 2 diabetes (T2DM) has also increased, and is predicted to rise further [2]. There is also an emerging consensus that T2DM and obesity are part of the same disease spectrum; as such, the term ‘metabolic syndrome’ is used to describe these overlapping pathophysiological processes [3]. Men with obesity, the metabolic syndrome, and T2DM have low total and free testosterone and low sex hormone-binding globulin (SHBG). Conversely, the presence of low testosterone and/or SHBG predicts the development of the metabolic syndrome and T2DM. Visceral adiposity, which is present in men with low testosterone, the metabolic syndrome, and/or T2DM acts through pro-inflammatory factors. These inflammatory markers contribute to vascular endothelial dysfunction with adverse sequelae such as increased cardiovascular disease (CVD) risk and erectile dysfunction (ED). This review focuses on the multidirectional impact of low testosterone levels associated with obesity and the metabolic syndrome and its effects on erectile dysfunction and CVD risk in men with T2DM [4].

Androgens play an important role not only in sex differentiation and development, but also in regulating the metabolism of glucose, proteins, lipids and some inflammatory factors, all of which might have a great influence on insulin sensitivity. It is well known that reduced levels of total testosterone (TT) in middle-aged or older men may contribute to abdominal obesity, increased insulin resistance (IR), and diabetes mellitus. At least six large prospective clinical trials have suggested that reduction of TT predicted the increasing incidence of T2DM [5,6]. Low concentrations of endogenous androgens have been linked with IR, which is an important upstream driver for metabolic abnormalities such as hyperglycemia, hypertension, or dyslipidemia, and increased cardiovascular risk [7,8]. Moreover, results from the Massachusetts Male Aging Study suggest that a low concentration of testosterone might play a role in the development of IR and subsequent T2DM [9]. Keating *et al*. [10] recently demonstrated that androgen deprivation therapy is associated with an increased incidence of diabetes and cardiovascular diseases (CVD). On the other hand, administration of testosterone to hypogonadal middle-aged men improved insulin sensitivity and glucose homeostasis. Studies in laboratory animals support the hypothesis that diabetes has detrimental effects on testicular function; reduction in both Leydig cell number and testosterone secretion has been reported [11].

As it has been previously shown, patients with T2DM have a frequent occurrence of hypogonadotrophic hypogonadism as reflected in low plasma concentrations of testosterone and inappropriately low luteinizing hormone (LH) and follicle-stimulating hormone (FSH) [12]. In view of the increasing incidence of obesity and T2DM in younger populations and in children, the occurrence of this defect in younger populations needs to be investigated since such an abnormality would affect their sexual function, reproductive capability, and their quality of life during their peak reproductive years. In addition, the lack of testosterone would also potentially promote further weight gain and loss of skeletal muscle and would thus promote IR [13,14].

In the European Male Aging Study database of 3,369 men between the ages of 40 and 79 years, 3 sexual symptoms (poor morning erections, low sexual desire, and ED) had a syndromal relationship with decreased testosterone levels [15]. Moreover, in the European Male Aging Study, low serum testosterone was more frequent in men with comorbidities such as obesity, metabolic syndrome, and T2DM. In studies from diabetic clinics, total, bioavailable, and free testosterone levels were low in men with T2DM [16,17]. When comparing testosterone levels in men with and without ED and T2DM, these investigators found significantly lower serum bioavailable testosterone (*P* =0.006) and free testosterone (*P* =0.027) levels in men with ED, but there was no significant difference in total testosterone levels. The lower the serum testosterone appears, the greater the severity of ED is suggested [16]. Corona *et al*. [18] evaluated 1,200 men with ED and reported that 16% had T2DM. Serum total testosterone levels were below the reference range (300 ng/dl or, 10.4 nmol/l) in 24.5% of men with diabetes versus 12.6% of non-diabetic subjects (*P* =0.0001) after adjustment for age and body mass index (BMI). In addition, hypogonadism in men with T2DM was associated with decreased sexual desire, more symptoms of depression, and lower LH levels.

Multiple epidemiological studies have shown that low testosterone levels are associated with and predict the future development of T2DM and the metabolic syndrome although this relationship is confounded by the association of total testosterone with SHBG, free testosterone remains associated with measures of IR and T2DM in some, but not all studies. Although the link between low testosterone levels and IR is not solely a consequence of adiposity, the study by Grossmann *et al*. suggests that a substantial component is mediated through its association with body fat, in particular abdominal visceral adipose tissue. This testosterone-fat relationship is bi-directional, as both weight loss and testosterone therapy increase testosterone levels, reduce fat mass, and decrease IR [19].

A meta-analysis by Ding *et al.* [20] analyzed 20 cross-sectional studies with a total of 850 men with diabetes and 2,000 non-diabetic controls. Individual studies included in this meta-analysis were small, with a mean of 39 diabetic patients per study (range, 8 to 155), and consisted of highly selected populations and convenience samples. Nevertheless, total testosterone levels were consistently lower in diabetic men compared with non-diabetic controls in all individual studies, with a mean pooled difference of 2.66 nmol/l (95% confidence interval (CI), 3.45 to 1.86). The difference in total testosterone was less, _1.61 nmol/l (95% CI,_2.56 to_0.65), but was still significant after adjustment for age and crude measures of body fat, such as BMI and waist circumference. These findings were confirmed by a more recent meta-analysis of 28 cross-sectional studies including 1,822 men with diabetes and 10,009 non-diabetic controls by Corona *et al.* [21]; total testosterone was lower in men with diabetes compared with controls (mean difference, 2.99 nmol/l (95% CI, 3.59 to 2.40)), and diabetes remained associated with lower total testosterone levels independent of age and BMI (adjusted r_ = 0.568; *P*_ = 0.0001) [6]. However, neither Ding *et al*. [20] nor Corona *et al*. [21] analyzed the association of free testosterone levels with diabetes, due to the lack of reliable data.

However, a cross-sectional study from the Third National Health and Nutrition Examination Survey (NHANES) group including 1,413 adult men, 101 of which had diabetes, showed that men in the lowest fertile of calculated free testosterone, adjusted for age, ethnicity, and adiposity, were more likely to have prevalent diabetes (odds ratio, 4.12; 95% CI, 1.25 to 13.55; *P* _ 0.04) [22]. In addition, a recent cross-sectional analysis of 1,292 men from the Norfolk population found that the 156 men with a hemoglobin A1c (glycosylated hemoglobin; HbA1c) of at least 6.5% or self-reported diabetes had, compared with men with an HbA1c no greater than 5%, a 2.4 nmol/l lower circulating total and a 30 pmol/l lower free testosterone level, respectively (*P* _ 0.001) [23]. Other recent cross-sectional studies included larger numbers of men with diabetes (between 100 and 580), but were limited by the absence of a non-diabetic control group. These studies from Australia [24], the United Kingdom [25], and the United States [26] consistently showed that 30 to 50% of aging, obese men with diabetes, in the absence of known testicular or pituitary pathology, have low total or free testosterone, at least relative to reference ranges based on healthy young men. The degree of hypotestosteronemia, however, was moderate, with mean total testosterone levels ranging from 10.5 to 12.7 nmol/l [24-26]. In these men, similar to what is found for men without diabetes, the prevalence of low testosterone increases with age and adiposity, so that in one study, two thirds of men who were older than 65 years or obese had low testosterone [24].

### Aim of the study

The aim of study was to show the influence of testosterone replacement therapy (TRT) on obesity, HbA1c level, arterial hypertension and dyslipidemia in patients diabetes mellitus with androgen deficiency.

## Methods

One hundred and twenty-five male patients with diabetes mellitus were tested; subject enrollment was recorded for the period from 2010 until the end of 2013, at the LTD ‘National Institute of Endocrinology’ (Tbilisi, Georgia). The study was approved by the Ethics Committee of this institute. Written informed consent was obtained from all participants. Inclusion criteria were: T2DM, patients with BMI 27.0 to 48.0 kg/m^2^, patient age range 30 to 65 years, positive screening questionnaire for androgen deficiency in males [26]. Exclusion criteria were: diabetes mellitus type 1, hyperprolactinemia, BMI <27 kg/m^2^ and >48 kg/m^2^, kidney and liver disease, adenoma of prostate grade II to III, secondary hypogonadism, treatment by testosterone or testosterone stimulation therapy 3 months before screening. Treatment with anti-obesity drugs within 3 months prior to informed consent, uncontrolled hyperglycemia with a glucose level >240 mg/dl (>13.3 mmol/l), any previous (or planned within next 12 months) bariatric surgery (open or laparoscopic) or intervention (gastric sleeve), current treatment with systemic corticosteroids at time of informed consent or pre-planned initiation of such therapy (note: inhaled use of steroids (for example, for asthma/chronic obstructive pulmonary disease (COPD)) is not an exclusion criterion, as this does not cause systemic steroid action). Congestive heart failure of the New York Heart Association (NYHA) class III or IV, acute or chronic metabolic acidosis (present condition in patient history), hereditary galactose intolerance, alcohol or drug abuse within the 3 months prior to informed consent that would interfere with trial participation, acute coronary syndrome ≤6 weeks prior to informed consent, stroke or transient ischemic attack (TIA) ≤3 months prior to screening, change in dose of thyroid hormones and dyslipidemia therapy within 6 weeks before screening.

The following analyses were conducted: 1) anthropometric study: height and weight were evaluated in all subjects. BMI was calculated as weight (kg) divided by square of height (m^2^) and was expressed in kg/m^2^. Waist circumference was measured midway between the iliac crest and the lower margin of the 12th rib. Also, for measurement of supine blood pressure subjects were in a supine or semi-recumbent position for a minimum of 5 minutes before the blood pressure measurement; after that blood pressure measurement was recorded to the nearest 2 mmHg mark on the manometer, which was conducted using a calibrated manometer; 2) biochemical measurements were made after a 10 to 12-hour fast: venous blood samples were obtained from 9:00 to 11:00 am and stored at 4°C; HbA1c, liver function tests, lipid profile, free testosterone, leptin, prostate specific antigen (PSA), thyroid stimulating hormone (TSH), FSH, LH; all hormone assays were performed by the fully automated ELISA analyzer Elisys Uno (HUMAN Diagnostics, Wiesbaden, Germany); 3) validation of a screening questionnaire for androgen deficiency in males (adapted from Morley JE, et al. *Metabolism.* 2000; 49(9):1239–1242); 4) ultrasonography of the abdomen and prostate were performed with a Siemens Acuson Antara (Serial #CA94043); 5) electrocardiography (ECG).

Eighty-five subjects, with age range 41 to 65 years and BMI from 27.0 to 48.0 kg/m^2^, were randomized in a placebo-controlled study. According to the laboratory and clinical conditions, we divided patients into two groups: 1) a treatment group; 2) a placebo group. In the first group we used diet, physical activity (lifestyle intervention implies reduced calorie diet - the reduction of daily calorie intake to 1800 to 2000 calories – and this was selected individually), patient’s antidiabetic therapy and TRT (testosterone undecanoate 250 mg/ml intra-muscularly once every 3 months). In the second group we used diet, physical activity (lifestyle intervention implies reduced calorie diet - the reduction of daily calorie intake to 1,800 to 2,000 calories – and this was selected individually), patient’s antidiabetic therapy and placebo.

Shapiro-Wilk test was used to determine normality of data distribution. The results were presented as mean ± standard deviation (SD) and 95% confidence interval (CI 95%) for normally distributed data. Non-normally distributed data were displayed as median with range (minimum to maximum values). Differences between the study group and control group patients were measured using independent sample *t*-test for normally distributed data and Mann–Whitney *U*-test for non-normally distributed variables. Pearson’s (r) correlation tests were used to determine the relationship between study parameters, depending on variable distribution. *P*-value <0.05 was considered statistically significant. Statistical analysis was performed using the SPSS 15.0 software package (SPSS, Inc., Chicago, IL, USA).

## Results

**Figure 1:**
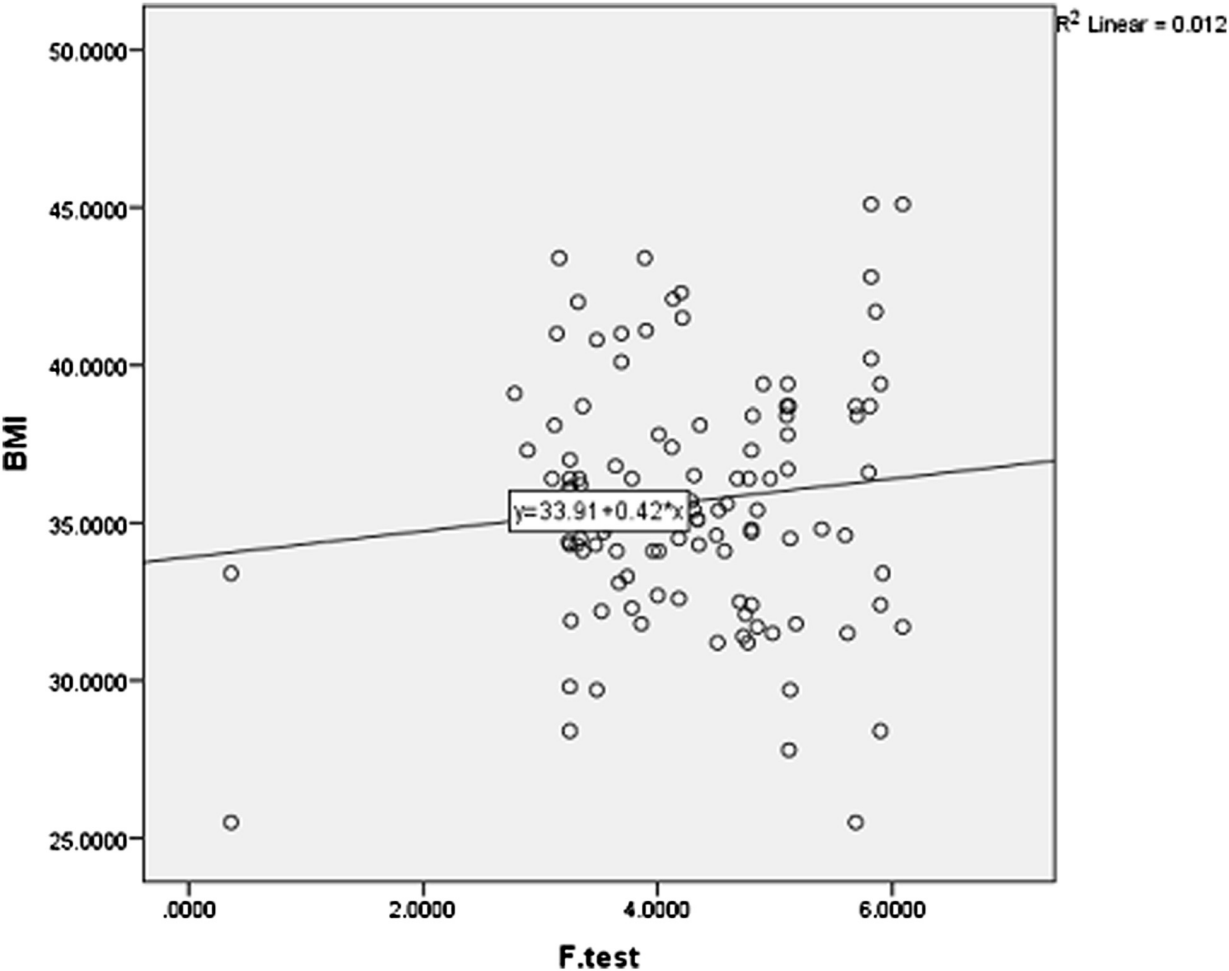
**Correlation between body mass index (BMI) and free testosterone.**

In all investigated patients, increased leptin levels were observed: average leptin level was 22.31 ng/ml (normal range: 0.2 to 5.0 ng/ml) (Table 1). Average BMI level was 35.38 kg/m^2^ (Table 1), and also decreased was the free testosterone level. Average free testosterone level was 4.85 ng/dl (Table 1) (normal range:.5.8 to 36 ng/dl) and was inversely correlated with the degree of obesity (BMI) (Figure 1) (r = 0.10), but there was no correlation between free testosterone and waist circumference (r = 0.56). Average HbA1c level was 8.35% (Table 1). Also, abnormal lipid profile was observed (Table 1). All patients had diabetes mellitus and 59 patients had arterial hypertension (Table 1).

**Table 1:** **Descriptive characteristics**

**Characteristic**	**Average pooled (n =85)**
Age	49.7
BMI	35.83
WC (cm)	119.69
SP (mmHg)	130.11
DP (mmHg)	86.13
F.test	3.82
HbA1c	8.35
Leptin	22.31
TC	209.2
TG	183.88
HDL	54.00
LDL	101.25
DM	85
AH	59

Data are expressed as mean ± SD. AH: arterial hypertension; BMI: body mass index; Chol: cholesterol; DM: diabetes mellitus; DP: diastolic blood pressure; F.test: free testosterone; HbA1c: glycosylated hemoglobin; HDL: high-density lipoprotein cholesterol; LDL: low-density lipoprotein cholesterol; SP: systolic blood pressure; TC: total cholesterol; TG: triglyceride; WC: waist circumference.

The patients were randomized in 2 1-sided blinded groups: 1) in the first group we had 42 patients (Table 2); 2) while in second group we had 43 (Table 2). In the first group, existing antidiabetic therapy remained unchanged, but diet, physical activity and TRT were added. For these patients we used injection of testosterone undecanoate once every 3 months. In the second group, existing anti-diabetic therapy remained unchanged, but diet, physical activity and placebo were added.

**Table 2:** **Descriptive characteristics**

**Characteristic**	**Group I average results (n =43)**	**Group II average results (n =42)**	***P*** **-value**
Age	49.8	49.6	0.98
BMI	35.65	36.0	0.98
WC (cm)	118.64	120.26	0.98
SP (mmHg)	128.43	130.12	0.98
DP (mmHg)	85.26	86.05	0.98
F.test	3.85	3.80	0.98
HbA1c	8.26	8.43	0.98
Leptin	21.46	23.19	0.98
TC	211.17	207.28	0.98
TG	185.6	182.21	0.98
HDL	55.50	54.0	0.98
LDL	102.95	99.58	0.98

Data are expressed as mean ± SD. BMI: body mass index; Chol: cholesterol; DP: diastolic blood pressure; F. test: free testosterone; HbA1c: glycosylated hemoglobin; HDL: high-density lipoprotein cholesterol; LDL: low-density lipoprotein cholesterol; SP: systolic blood pressure; TC: total cholesterol; TG: triglyceride; WC: waist circumference.

**Figure 2:**
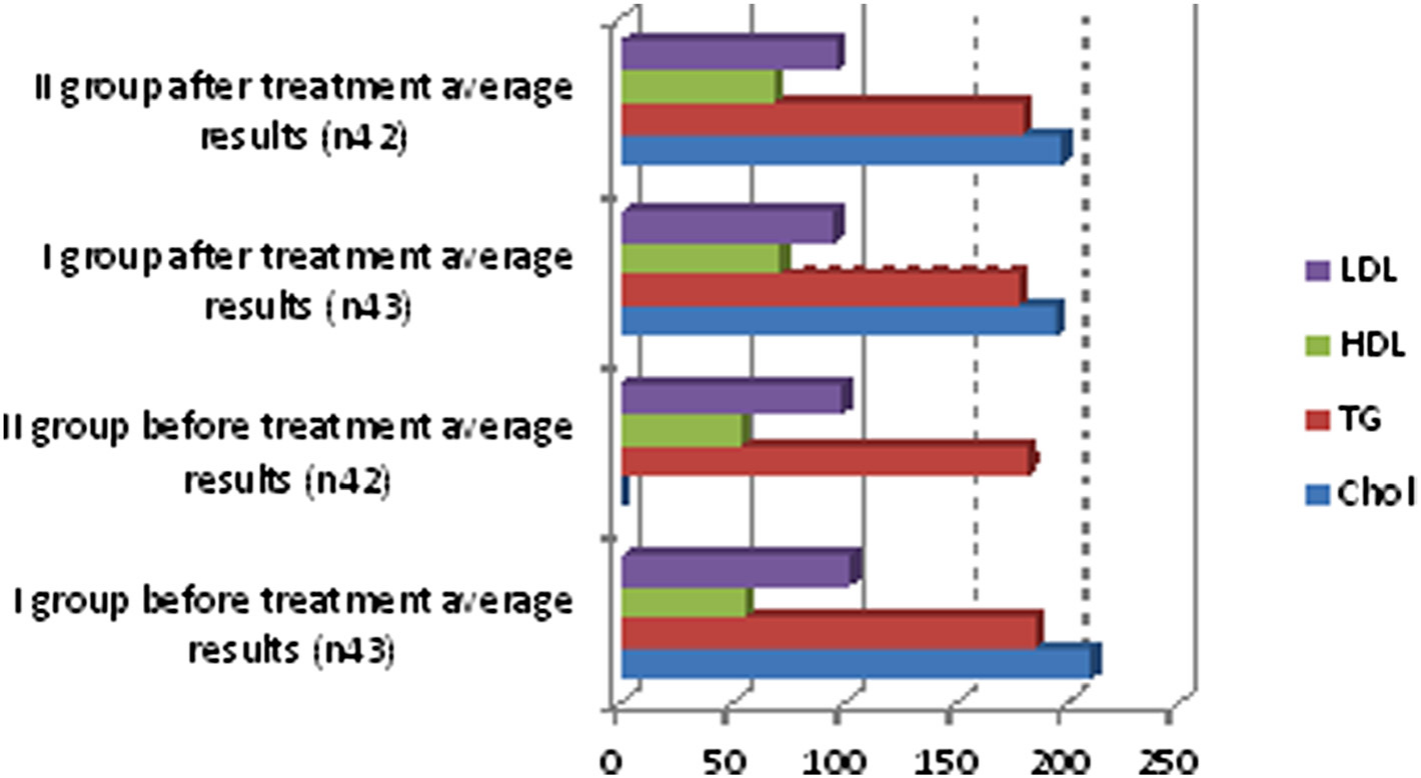
**Descriptive characteristics.**

**Figure 3:**
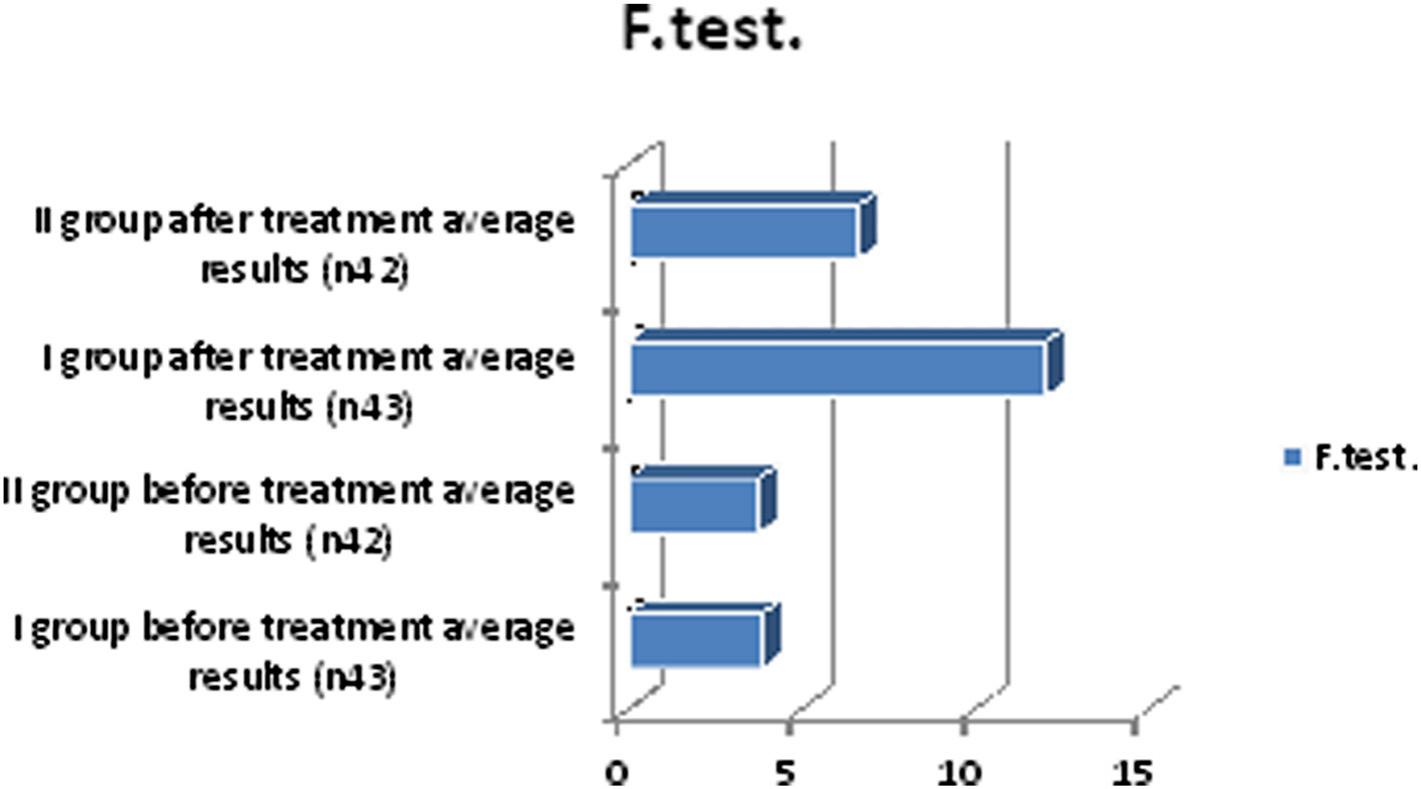
**Descriptive characteristics: free testosterone level before treatment and after treatment in both groups.**

**Figure 4:**
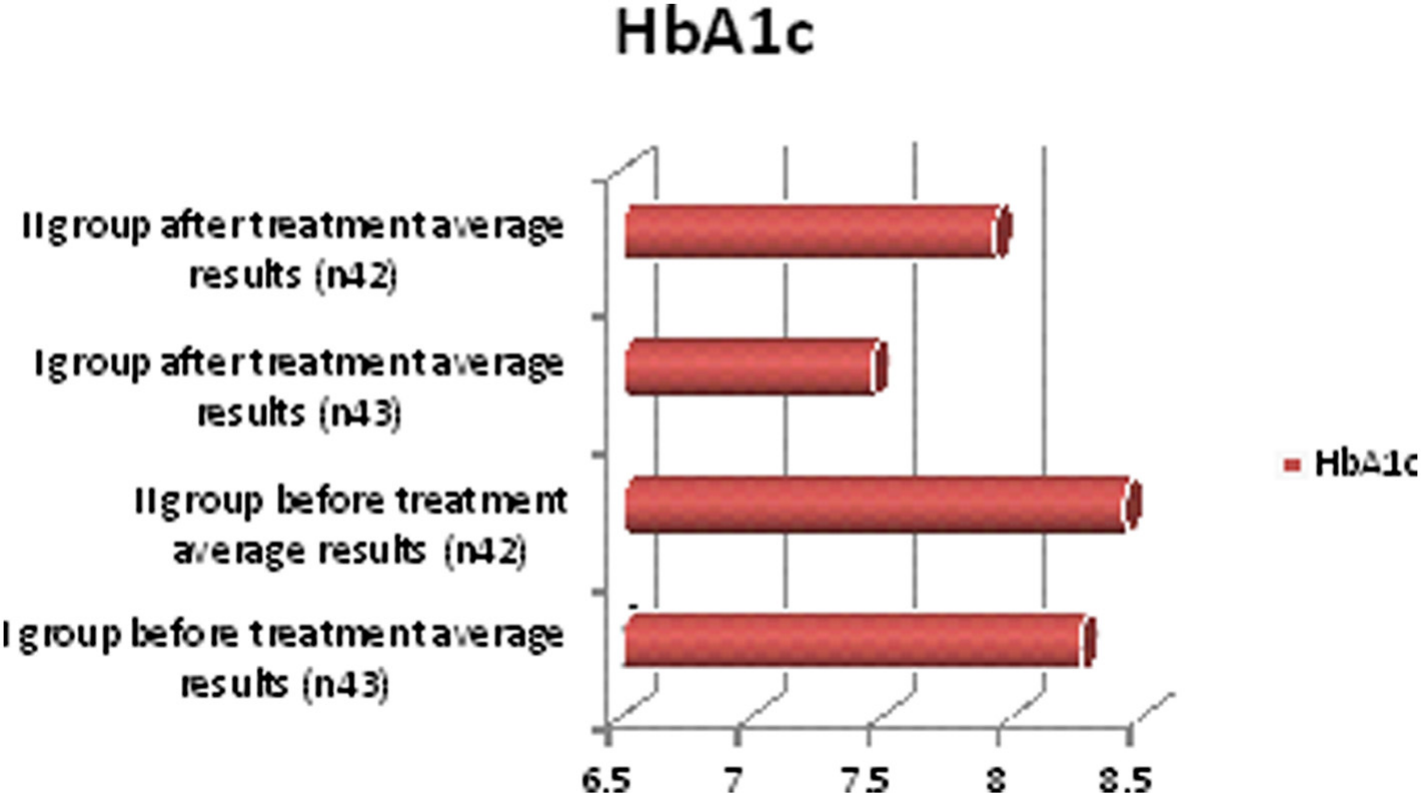
**Descriptive characteristics: glycosylated hemoglobin before treatment and after treatment in both groups.**

**Figure 5:**
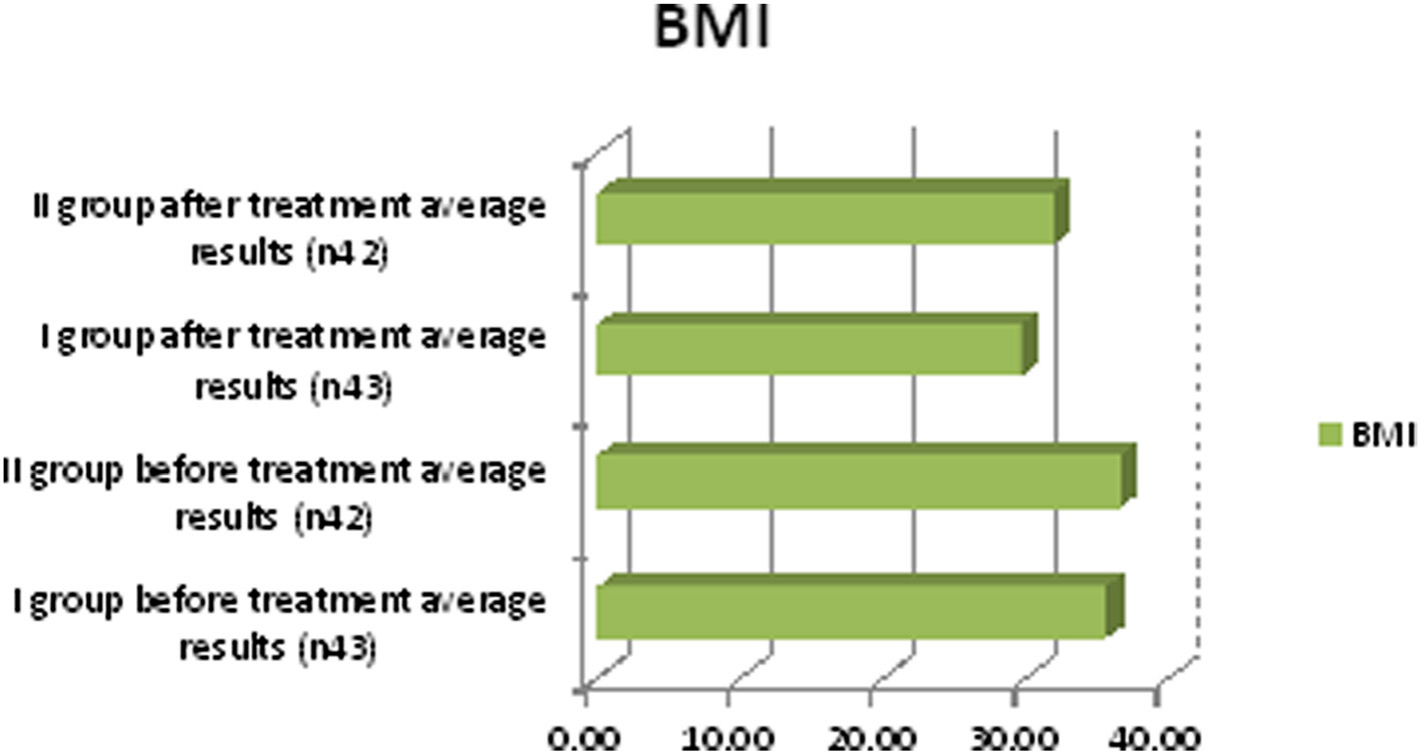
**Descriptive characteristics: body mass index (BMI) before treatment and after treatment in both groups.**

**Figure 6:**
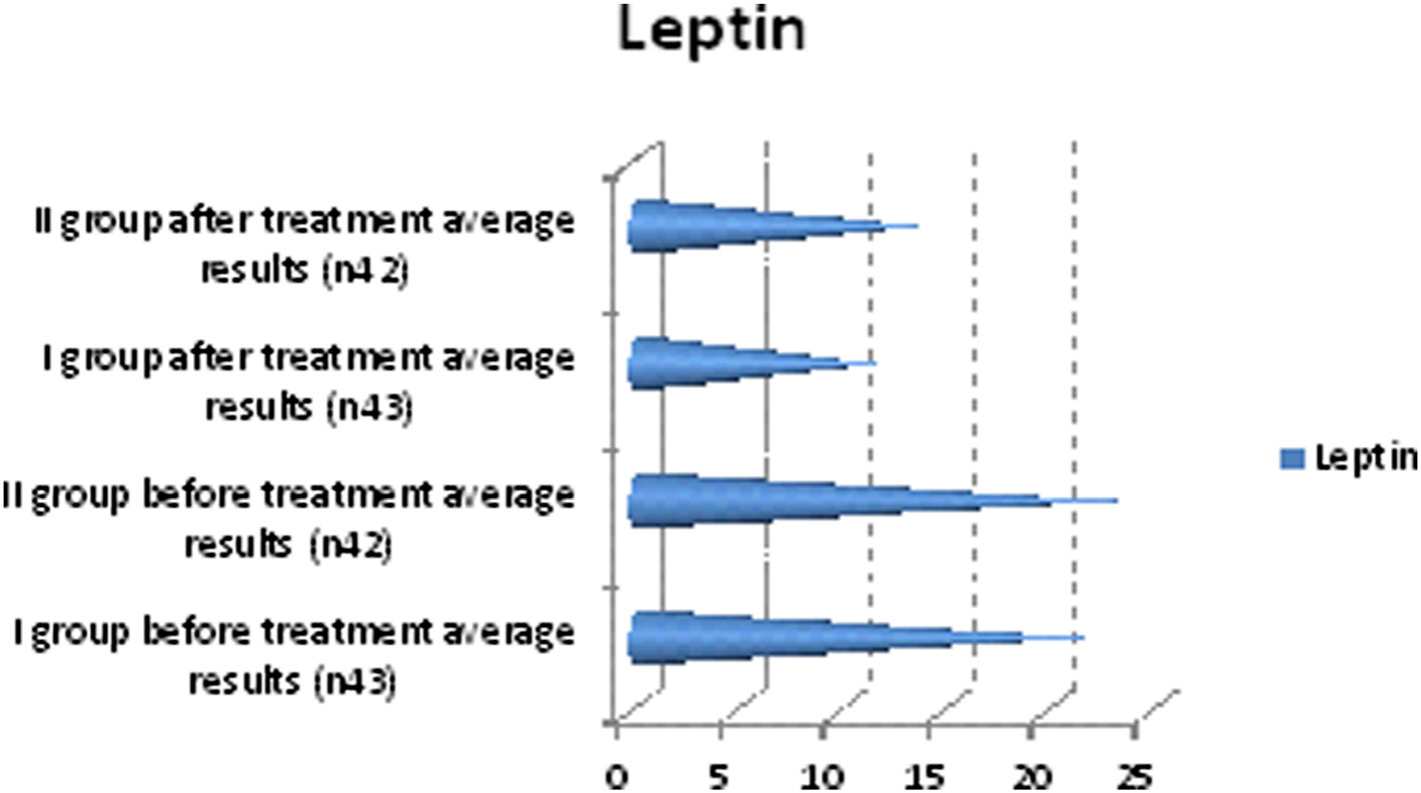
**Descriptive characteristics: leptin level before treatment and after treatment in both groups.**

**Figure 7:**
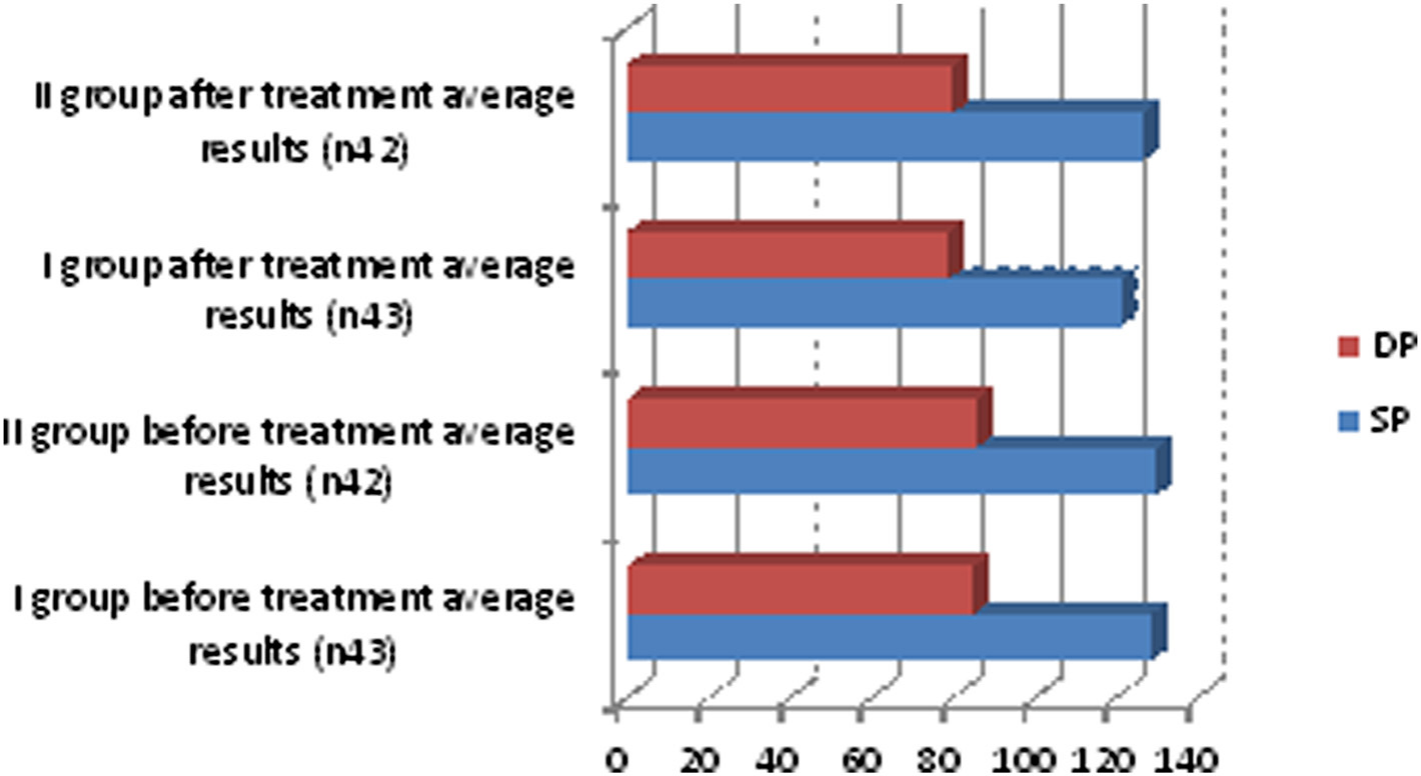
**Descriptive characteristics: systolic and diastolic blood pressure before treatment and after treatment in both groups.**

We observed in which group we had the best outcomes and after 6 months of treatment we repeated the diagnostic assessments. These observations yielded some positive results for lipid profiles: cholesterol, triglyceride and low-density lipoprotein (LDL) cholesterol levels decreased, and high-density lipoprotein (HDL) cholesterol increased in both groups but more clinically significant results were shown in the first group (Table 3, Figure 2). Free testosterone level increased in all groups but the clinically significant results were shown in group I (Table 3, Figure 3), where testosterone undecanoate was used. HbA1c decreased in both groups but in group I we had the best results (Table 3, Figure 4). BMI decreased in both groups but a greater reduction was seen in group I (Table 3, Figure 5). Leptin level after treatment was approximately the same in both groups, but the relative best results were achieved in group I (Table 3, Figure 6). Blood pressure reduction was the same in both groups (Figure 7).

**Table 3:** **Descriptive characteristics**

	**Group I before treatment average results (n =43)**	**Group I after treatment average results (n =43)**	**Group II before treatment average results (n =42)**	**Group II after treatment average results (n =42)**
BMI	35.65	29.68	36.60	31.88
F.test	3.85	12.06	3.8	6.64
HbA1c	8.26	7.46	8.43	7.93
Leptin	21.71	11.64	23.19	13.93
Chol	211.17	194.64	207.28	198.02
TG	185.6	178.31	182.21	180.23
HDL	55.5	71.11	54	68.44
LDL	102.95	95.62	99.58	96.3

Data are expressed as mean ± SD. BMI: body mass index; Chol: cholesterol; F.test: free testosterone; HbA1c: glycosylated hemoglobin; HDL: high-density lipoprotein cholesterol; LDL: low-density lipoprotein cholesterol; TC: total cholesterol; TG: triglyceride.

## Discussion

Serum testosterone, HbA1c, HDL cholesterol, triglyceride concentrations, BMI, and hypertension improved in both treatment groups after 26 weeks of treatment. We have shown that TRT improves IR and glycemic control in hypogonadal men with diabetes. TRT reduced the HOMA-IR index, indicating an improved fasting insulin sensitivity. This study demonstrates that intramuscular TRT in hypogonadal men with T2DM improves IR, the central biochemical defect associated with these conditions. Although an improvement in IR could be expected to result in better glycemic control, 6 months of treatment demonstrated reduction of HbA1c. Small-scale studies of testosterone treatment in men with metabolic syndrome or T2DM and marginal low or normal testosterone levels showed improvement in glycemic control. The low testosterone levels might have induced a vicious circle, since low testosterone levels induce an increase in visceral obesity and severity of cardiovascular risk factors compared with the metabolic syndrome. Studies have shown that hypogonadism is associated with dyslipidemia, but there are only limited data supporting the effect of testosterone on lipids. Two studies reported a small rise in HDL cholesterol but no effect on TC or LDL cholesterol in hypogonadal men [27].

Furthermore, after TRT, total cholesterol, LDL cholesterol, triglyceride had reduced and HDL cholesterol increased by 16.53 mg/dl, 7.33 mg/dl, 7.29 mg/dl and 15.61 mg/dl respectively, which are consistent with other TRT trials’ results [28]. Men with diabetes have significantly lower serum testosterone concentration than non-diabetic men. Low concentrations of endogenous androgens have been linked with increased cardiovascular risk; the increased risk for CVD in diabetic men could be partially mediated through low concentrations of testosterone. Increased age is one of the strongest predictors for coronary artery disease. The Telecom Study demonstrated a significant decrease in testosterone concentration with each decade of life [29].

It has been shown that lower levels of testosterone in men are associated with higher blood pressure. The first study to demonstrate a favorable effect of testosterone treatment on blood pressure in abdominally obese men was published by Marin *et al*. [30]. Another study investigating the effects of testosterone treatment of men with osteoporosis found also a beneficial effect on blood pressure levels. In a study of 122 men receiving treatment with parenteral testosterone undecanoate over 15 months, both systolic and diastolic blood pressure decreased [31]. The maximum effect was achieved after 9 months of testosterone administration.

Furthermore, leptin also may have some role in this recovery. Leptin is an adipose tissue hormone with so-called ‘double action’: it affects receptors on Leydig cells, lowering testicular testosterone secretion. In addition, leptin suppresses pituitary LH secretion. Hypogonadal diabetic men have increased leptin levels and, as it has been shown, TRT reduces leptin levels in addition to the independent effects of weight loss in lowering leptin levels. A limitation of this study is the relatively small samples of subjects involved that can limit the generalizability of the study.

## Conclusions

Our study demonstrated that it is possible to break the metabolic vicious circle discussed above by raising testosterone levels in diabetic men with androgen deficiency [32]. In addition to traditional CVD risk factors, novel risk factors are also inversely related to testosterone levels. Re-instituting physiological levels of testosterone in hypoandrogenic men, as our small study has shown, has an important role in reducing the prevalence of diabetic complications, but large-scale randomized placebo-controlled trials are needed to fully evaluate this.

## Abbreviations

AH: Arterial Hypertension
BMI: Body mass index
CI: Confidence interval
COPD: Chronic obstructive pulmonary disease
CVD: Cardio-vascular disease
DP: Diastolic pressure
ECG: Electrocardiogram
ED: Erectile dysfunction
FSH: Follicle stimulation hormone
HbA1c: Glycosylated hemoglobin A1
HDL: High density lipoprotein-cholesterol
HOMA: IR
IR: Insulin resistance
LDL: low-density lipoprotein
LH: Luteinizing hormone
NYHA: New York Heart Association
PSA: Prostate specific antigen
SD: Standard deviation
SHBG: Sex-hormone binding globulin
SP: Systolic pressure
TC: Total cholesterol
TG: Triglycerides
TIA: Transitory ischemic attack
TRT: Testosterone replacement therapy
TSH: Thyroid stimulating hormone
TT: Total testosterone
T2DM: Type 2 diabetes mellitus
WC: Waist circumference
